# Short and longer-term psychological consequences of Operation Cast Lead: documentation from a mental health program in the Gaza Strip

**DOI:** 10.1186/1752-1505-6-8

**Published:** 2012-10-23

**Authors:** Augusto E Llosa, Germán Casas, Hélène Thomas, Angels Mairal, Rebecca F Grais, Marie-Rose Moro

**Affiliations:** 1 Epicentre, 8 rue Saint Sabin, Paris, 75011, France; 2 School of Medicine, Los Andes University, Carrera 7 N 116-05, Bogotá, Colombia; 3 Médecins Sans Frontières, 8 rue Saint Sabin, Paris, 75011, France; 4 Medecins Sans Frontieres, El Hajaj Ibn Youssuf Street, Shufat Main road, Jerusalem; 5 Cochin Hospital, Université Paris Descartes, Unité INSERM 669, Paris, 75014, France

**Keywords:** Mental health, Psychological, PTSD, Internally displaced persons, Refugees, Conflict, War, Cast lead, Palestinian, Gaza

## Abstract

**Background:**

There is growing recognition of the psychological impact of adversity associated with armed conflict on exposed civilian populations. Yet there is a paucity of evidence on the value of mental health programs in these contexts, and of the chronology of psychological sequelae, especially in prolonged conflicts with repeated cycles of extreme violence. Here, we describe changes in the psychological profile of new patients in a mental health program after the military offensive Cast Lead, in the context of the prolonged armed conflict involving the Gaza Strip.

**Methods:**

This study analyses routinely collected program data from a Médecins Sans Frontières mental health program in the Gaza Strip spanning 2007–2011. Data consist of socio-demographic as well as clinical baseline and follow-up data on new patients entering the program. Comparisons were made through Chi square and Fisher’s exact tests, univariate and multivariate logistic and linear regression.

**Results:**

PTSD, depression and other anxiety disorders were the most frequent psychopathologies, with 21% having multiple diagnoses. With a median of nine sessions, clinical improvement was recorded for 83% (1122/1357), and more common for those with separation anxiety, acute and posttraumatic disorders as principal diagnosis (855/1005), compared to depression (141/183, p<0.01). Noted changes proximal to Operation Cast Lead were: a doubling in patient case load with a broader socio-economic background, shorter interval from an identified traumatic event to seeking care, and a rise in diagnoses of acute and posttraumatic stress disorders. Sustained changes included: high case load, more distal triggering events, and increase in diagnoses of other anxiety disorders (especially for children 15 years and younger) and depression (especially for patients 16 years and older).

**Conclusion:**

Evolving changes in patient volume, diagnoses and recall period to triggering events suggest a lengthy and durable effect of an intensified exposure to violence in a context of prolonged conflict. Our findings suggest that mental health related humanitarian relief in protracted conflicts might need to prepare for an increase in patients with changing profiles over an extended period following an acute flare-up in violence.

## Background

Prolonged exposure to violence increases the risk of accumulation of major traumatic events and daily life stressors, including physical and economic insecurity, all of which have negative mental and psychosocial consequences [1-4]. A high number of accumulated traumatic life events, economic pressure, and elevated prevalence of depression, anxiety and posttraumatic stress disorder (PTSD) have been found among adults and children in the Gaza Strip [5-9].

Close to a million and a half people live in the 360 square kilometers that make up the Gaza Strip [10]. Crowded, confined and impoverished, the strip’s population has faced increasing tension, war and deteriorating living conditions in the last decade. Economic pressure, political changes, and restrictions and violence related to the Al Aqsa intifada, and Israeli military involvement have contributed to noted de-development, increased dislocation and exposure to violence [9,11,12]. Shelling during the military offensive Operation Cast Lead (OCL) from 27 December 2008 to 18 January 2009, resulted in between 1,166-1,417 deaths, including 300 children, many more injured, and the destruction of homes and infrastructure, massive displacement and financial loss in the Gaza Strip. This intense, prolonged bombardment and military occupation exposed the community to the conflict to an unprecedented degree, adding economic and psychological stressors while disrupting existing social support networks [13-15].

The Mental Health Services Organization Plan was adopted by the Palestinian Ministry of Health (MoH) in 2004. Besides an MoH run psychiatric hospital, the Gaza Community Mental Health Program and NGOs provide psychological and psychosocial support to this population. After OCL, however, mental health services were temporarily disrupted. The international medical humanitarian organization Médecins Sans Frontières/Doctors without Borders (MSF) has provided free medical support to the population of Occupied Palestinian Territory since 1989. The mental health program in Gaza opened in 2000 and included medical care, social worker assessment and support, psychological evaluation and psychotherapy. Consenting civilian victims of the conflict residing in Gaza, at least one year of age and in need of treatment were eligible for free care. Patients with chronic psychiatric illnesses (schizophrenia, mental retardation, personality disorders) were referred to the Ministry of Health psychiatric hospital for specialized treatment. Therapeutic consultations took into account the social and cultural context, utilized local therapists or translators, [16,17] lasted typically 8–12 sessions, and were guided by principles of interpersonal psychotherapy [18-20]. While additional rigorous assessments are still needed, there appears to be an overall benefit of appropriately targeted psychotherapy in war and other humanitarian crises [4,9,21-23].

Two prior analyses of the mental health project data provide details on the therapeutic approach and patient descriptions from the project’s opening in 2000 through 2006 [24] and 2005 through 2008 [21]. The present operational review aims to update this description through 2011 and explore potential changes in diagnoses, severity and treatment outcomes potentially associated with the intensification of the conflict during Cast Lead. This exploratory analysis was intended as descriptive and hypothesis generating.

## Methods

This analysis draws from routinely collected information from the patient database of the MSF mental health program in Gaza Strip for files opened between 2007 and the program’s closure in 2011. Analysis includes patient baseline as well as follow-up data.

Semi-structured interviews with standardized questionnaires were used by mental health officers to collect information on socio-demographics, main complaints, presenting symptoms and traumatic event history. Diagnoses and severity at baseline and final session were assigned by MSF psychologists or psychiatrists according to DSM-IV-TR criteria and in accordance to guidelines laid out by the supervising psychiatrist [25]. For simplification and to improve cultural specificity of diagnoses, focus group discussions were conducted at the opening of the program. As a result, the PTSD category included complex-PTSD; [26] dysthymia was included in the depression category; and other anxiety disorders included generalized anxiety disorder, panic disorder, agoraphobia, social and specific phobia.

Disorder severity categories (mild, moderate, severe) considered symptom severity as well as psychological and social impairment. Following initial standardized assessment by mental health officers, where indicated, patients were referred to psychologist, psychiatrist or other medical doctor according to their need. Therapy was either individual, group or dyad (caregiver/child). After 8–12 sessions, the patients’ condition was re-assessed and therapy was either extended, finished or the patient was referred. Final outcome was qualitatively categorized (resolved, improved, unchanged, worsened) taking into account the patient’s perception of their state and the treating psychologist’s evaluation of changes in psychological and social functioning, symptom severity, resilience and coping strategies. Reasons for discontinuation of treatment were recorded. Delay to seeking care was computed, where relevant, as the number of weeks between an identified traumatic event and time when care was sought. Its association with the timing of OCL was explored as potential indicator of impact of this particularly violent period in the context of a sustained conflict.

Age was categorized as 15 years and younger (child or young adolescent) and above 15 years (older adolescent or adult), corresponding to the differences in the therapeutic intervention process for patients in MSF programs relating to these age categories. Specific OCL exposures were not recorded in the database, but the timing of the patients’ initial assessment was later categorized in reference to the war period (number of months before 27 December 2008 or after 17 January 2009). Returning patients (estimated as less than 5% from operational summaries) were not excluded. Categorical variables were compared by Chi square (*χ*^2^) and Fisher’s exact tests. Odds ratios (OR) and 95% confidence intervals (95% CI) were computed through logistic regressions for dichotomous outcomes. Coefficients (ß) and p-values for ordinal outcomes were computed through linear regression models. Regression coefficients and OR are from univariate models unless otherwise specified. A p-value lower than 0.05 was considered statistically significant. As the analysis was exploratory and all available data were included, no a priori sample size calculation was performed.

Anonymized patient data were entered into Epidata (Odense, Denmark) and analyzed with STATA 10.1 (College Station, Texas, USA). The program was authorized by local authorities and patients were informed about the use of data in research. Privacy and confidentiality of patients were ensured during the treatment and after the conduct of the analysis. This analysis met the criteria for review of program monitoring data and for exemption from the MSF Ethics Review Board.

## Results

This analysis included 1357 evaluable of 1377 (98.5%) MSF mental health clinic patients whose treatment started between January 2007 through July 2011. On average during this period, 25 patients each month started treatment; highest monthly averages were noted in the two years following OCL. Patients’ age ranged from three to 70 years, with a median of 13 and inter-quartile range (IQR) = 9–24 years; 63% (n=738) were 15 years of age or younger and 55% were female. From 2007 through 2011 the proportion of patients 15 years and younger increased, OR = 0.73 (95% CI = 0.66-0.80), as did, among those at least 16 years of age, the proportion of women, OR = 1.37 (1.25-1.50) (Table 1A).

**Table 1:** Patient socio-demographic and referral characteristics by year of entry into the MSF program

**Year**	**2007**		**2008**		**2009**		**2010**		**2011**		**Total**	
**Demographic**												
New registrations, n	149		206		411		388		203		1357	
Monthly Average, n (SD)	12.4	(8.1)	17.2	(7.2)	34.3	(22.8)	32.3	(11.3)	28.9	(12.7)	24.7	(16.0)
Adult:child ratio	1.6		0.8		0.5		0.5		0.4		0.6	
<15 y male:female ratio	1.7		1.0		1.3		1.4		0.9		1.2	
>15 y male:female ratio	1.7		1.8		0.9		1.0		0.8		1.2	
**Socio-economic needs**												
High need, n (%)	78	(53.0)	119	(61.0)	231	(57.0)	251	(65.0)	143	(70.0)	822	(62.0)
Some need, n (%)	61	(42.0)	59	(30.0)	145	(36.0)	119	(31.0)	57	(28.0)	441	(33.0)
Needs covered, n (%)	7	(5.0)	17	(9.0)	31	(8.0)	14	(4.0)	3	(1.0)	72	(5.0)
Total, n (%)	146	(100)	195	(100)	407	(100)	384	(100)	203	(100)	1335	(100)
**Referral source**												
MSF outreach, n (%)	77	(52.0)	110	(54.0)	134	(33.0)	88	(23.0)	45	(22.0)	454	(34.0)
Local institution, n (%)	17	(11.0)	34	(17.0)	45	(11.0)	31	(8.0)	17	(8.0)	144	(11.0)
Self, n (%)	10	(7.0)	20	(10.0)	20	(5.0)	40	(10.0)	28	(14.0)	118	(9.0)
Ministry of Health, n (%)	12	(8.0)	0	(0)	1	(0.0)	2	(1.0)	0	(0)	15	(1.0)
Community/family, n (%)	25	(17.0)	41	(20.0)	209	(51.0)	223	(57.0)	108	(53.0)	606	(45.0)
Other, n (%)	8	(5.0)	0	(0)	1	(0)	4	(1.0)	5	(2.0)	18	(1.0)
Total, n (%)	149	(100)	205	(100)	410	(100)	388	(100)	203	(100)	1355	(100)

Monthly average for 2011 and total consider only active portions (January-July) of 2011.

Age not recorded for 5 patients.

Sex not recorded for 2 patients.

Socio-economic needs assessment results not recorded for 22 patients.

Referral source not recorded for 2 patients.

The majority (93%) of patients were rated as having high (n=822) or medium (n=441) socio-economic needs during the intake interview. Overall, the proportion of those with high socio-economic needs increased over time, OR 1.18 (1.08-1.30), except briefly after the war in 2009 when there was a greater proportion of new patients with medium to low socioeconomic needs compared to both 2008, OR = 1.19 (0.84-1.69), and 2010, OR= 1.44 (1.08-1.92) (Table 1B).

The majority of patients were referred by community/family (45%), MSF social workers (34%) or other institutions (11%). Community/family and self referrals increased over time compared to those from MSF social workers and other institutions, OR = 1.74 (1.57-1.92). This trend was most pronounced in 2009 following the war but continued in 2010 and 2011. (Table 1C).

Of 1357 patients seen in this period, 1305 (96%) expressed that they were exposed to conflict related violence. Overall, 1137 (84%) considered Israeli Defense Forces (IDF) the source of exposure with this proportion significantly increasing from 72-74% in 2007 and 2008 respectively to 92% in 2009 (*χ*^2^ = 49.4, p<0.001).

Information on traumatic life events was available for 1352 patients, of which 1263 (93%) reported at least one. On average patients 15 years and younger, reported one event per three life-years (0.33 per year) and those older than 15 reported one per decade lived (0.11 per year) (ß = 0.22, p<0.001) (Table 2). Adjusted for age, new patients were more likely to report a greater number of traumatic events after OCL (ß=1.0, p<0.001).

**Table 2:** Traumatic life events by age category for MSF patients, 2007-2011

**Traumatic life events by age category**	**<15 years**		**≥ 15 years**		**Total**	
	**N**	**(%)**	**N**	**(%)**	**N**	**(%)**
Sexual violence	6	(1)	9	(2)	15	(1)
Physical injury	123	(15)	250	(49)	373	(28)
Close family member killed	250	(30)	186	(37)	436	(32)
Close family member died from illness	51	(6)	60	(12)	111	(8)
Witness to murder or physical abuse	451	(54)	347	(69)	798	(59)
Received threats	379	(45)	271	(54)	650	(48)
Incarceration	35	(4)	52	(10)	82	(6)
Loss of property	458	(55)	201	(40)	659	(49)
Forced to flee	472	(56)	190	(38)	662	(49)
Break-up of nuclear family	77	(9)	46	(9)	123	(9)
Total patients	836	(100)	506	(100)	1352	(100)

Age not recorded for 5 patients.

Traumatic events not recorded for 5 patients.

**Figure 1:**
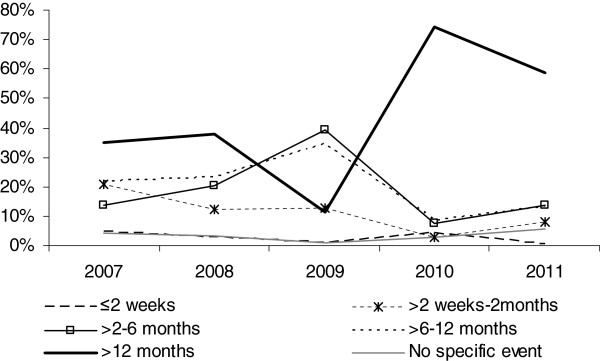
Time from triggering event to therapy by year for new patients in the MSF mental health treatment program in Gaza Strip, January 2007-July 2011.

For those with a stated triggering event (n=1295) the median delay to care was 7–12 months, and significantly longer for those 15 years and younger (ß= 0.33, p<0.001). Following OCL, in 2009 the median delay declined from 7–12 months to 3–6 months (ß = −0.41, p<0.001) and only 1% of patients did not identify an event associated with seeking psychological support. Delay significantly rose again in 2010 (ß= 1.1, p<0.001) with the majority of patients in 2010 (74%) and 2011 (59%) attributing a triggering event more than a year earlier, to the OCL time or before it (Figure 1).

Most common baseline symptoms noted in the younger age group were distress or anxiety followed by inhibition or withdrawal. For adults these were sadness or crying followed by distress or anxiety. One or two patients’ main complaints were reported at first visit. Most frequent for children were fear, sleep disturbance, bed-wetting and aggressiveness; for patients older than 15 years these were sleep disturbance, sadness, intrusive memories and anxiety (Table 3). After OCL, statistically significant increases (Fishers exact test) were noted in bed-wetting (p<0.05), sleep disturbance (p<0.05), and hyperactivity (p=0.001) among the younger patients, and in grief (p<0.01) and avoidance (p<0.05) for the older age group.

**Table 3:** Baseline clinical expression and main complaints of MSF program patients by age category, 2007-2011

	**Age category**			
	**<15 years**		**≥ 15 years**	
	**N**	**%**	**N**	**%**
**Clinical expression**				
Distress/anxiety	285	(34)	161	(32)
Inhibition/withdrawal	204	(24)	46	(9)
Sadness/crying	163	(19)	204	(41)
Unspecified	147	(17)	47	(9)
Aggression/anger	29	(3)	23	(5)
Agitation/logorrhea	13	(2)	20	(4)
Total	841	(100)	501	(100)
**Patient Complaints**				
Fear	568	(67)	93	(18)
Sleep disturbance	218	(26)	172	(34)
Enuresis	328	(39)	8	(2)
Sadness	56	(7)	163	(32)
Aggressivity	118	(14)	65	(13)
Intrusive memories	57	(7)	105	(21)
Anxiety/worries	56	(7)	100	(20)
Somatic complaints	21	(2)	90	(18)
Grief	18	(2)	65	(13)
Avoidance	35	(4)	20	(4)
Pain	8	(1)	38	(8)
Hyperactivity	43	(5)	2	(0)
Concentration problem	23	(3)	14	(3)
Other complaint	20	(2)	15	(3)
Speech problem	29	(3)	2	(0)
Learning disability	22	(3)	2	(0)
Weakness	5	(1)	11	(2)
Suicidal/risky behavior	4	(0)	8	(2)
Eating problems	7	(1)	4	(1)
Delusions/hallucinations	1	(0)	2	(0)
Total complaints	1637	(193)	979	(193)
Total patients	846	(100)	506	(100)

Clinical expression not recorded for 15 patients.

Patient complaints not recorded for 5 patients.

Anxiety disorders accounted for 83% (705/846) of diagnoses of patients 15 years and younger; depression and developmental disorders accounted for 6%. Compared to 2007–2008, the proportion of those diagnosed with PTSD rose in 2009, OR= 1.53 (1.03-2.27); and of other anxiety disorders in 2010, OR=1.85(1.17-2.90). Compared to 2008, intra-year trends in 2009 were a rise in diagnoses of acute stress disorder (ASD) first quarter, OR=5.38 (1.04-28.6); PTSD second quarter, OR=13.48 (4.6-39.3). Compared to the first six months of 2009, diagnoses of other anxiety disorders and depression increased in the second half of the year, OR=4.97 (2.29-10.80), in addition to communication disorders, attention deficit disorder and separation anxiety, albeit at lower frequencies. In 2010 and 2011 the proportion of new pediatric patients with PTSD and other anxiety disorders rose again to levels similar to before OCL.

Among patients older than 15, the most common diagnoses were PTSD, depression, other anxiety disorders, and ASD. Primary diagnoses of PTSD and ASD were more common in men OR = 1.98 (1.38-2.84) while depression and other anxiety disorders were more common in women OR= 1.91(1.34-2.72). In the first half of 2009, diagnoses of ASD and PTSD increased compared to the previous year, OR=2.70 (1.40-5.19). Compared to this period, in the second half of 2009 these two diagnoses proportionally decreased while depression and other anxiety disorders became more common, OR=4.97 (2.29-10.80). Patient diagnoses by age-group and year are shown in Tables 4 and 5.

**Table 4:** Main diagnosis for patients 15 years of age and younger by year of entry to MSF program

**Year**	**2007**		**2008**		**2009**		**2010**		**2011**		**Total**	
**Main diagnosis**	**No.**	**(%)**	**No.**	**(%)**	**No.**	**(%)**	**No.**	**(%)**	**No.**	**(%)**	**No.**	**(%)**
Acute stress disorder	1	(2)	2	(2)	6	(2)	6	(2)	3	(2)	18	(2)
PTSD	21	(37)	60	(54)	169	(62)	123	(48)	87	(59)	460	(54)
Other anxiety disorder	12	(21)	24	(21)	36	(13)	90	(35)	50	(34)	212	(25)
Depression	1	(2)	2	(2)	14	(5)	9	(4)	2	(1)	28	(3)
Brief psychotic disorder	0	(0)	0	(0)	2	(1)	0	(0)	0	(0)	2	(0)
Other psychotic disorder	1	(2)	0	(0)	0	(0)	0	(0)	0	(0)	1	(0)
Adjustment disorder	2	(4)	5	(4)	4	(1)	3	(1)	0	(0)	14	(2)
Learning disability	0	(0)	1	(1)	1	(0)	0	(0)	0	(0)	2	(0)
Communication disorder	0	(0)	1	(1)	9	(3)	3	(1)	0	(0)	13	(2)
Attention Deficit Hyperactivity	0	(0)	0	(0)	7	(3)	1	(0)	2	(1)	10	(1)
Eating disorder	0	(0)	1	(1)	1	(0)	0	(0)	0	(0)	2	(0)
Parent–child relationship	1	(2)	0	(0)	4	(1)	4	(2)	0	(0)	9	(1)
Separation Anxiety	0	(0)	0	(0)	8	(3)	7	(3)	0	(0)	15	(2)
Other diagnosis	2	(4)	3	(3)	1	(0)	0	(0)	0	(0)	6	(1)
No main diagnosis	0	(0)	0	(0)	2	(1)	0	(0)	0	(0)	2	(0)
Somatic disorder	1	(2)	1	(1)	1	(0)	0	(0)	0	(0)	3	(0)
Personality disorder	0	(0)	0	(0)	0	(0)	1	(0)	0	(0)	1	(0)
Distress /no disorder	7	(12)	7	(6)	6	(2)	6	(2)	3	(2)	29	(3)
Missing value	8	(14)	5	(4)	3	(1)	2	(1)	1	(1)	19	(2)
Total	57	(100)	112	(100)	274	(100)	255	(100)	148	(100)	846	(100)

**Table 5:** Main diagnosis for patients 16 years of age and older by year of entry to MSF program

**Year**	**2007**		**2008**		**2009**		**2010**		**2011**		**Total**	
**Main diagnosis**	**No.**	**(%)**	**No.**	**(%)**	**No.**	**(%)**	**No.**	**(%)**	**No.**	**(%)**	**No.**	**(%)**
Acute stress disorder	8	(9)	4	(4)	8	(6)	3	(2)	0	(0)	23	(5)
PTSD	35	(39)	46	(49)	72	(53)	31	(23)	15	(27)	199	(39)
Other anxiety disorder	6	(7)	8	(9)	13	(10)	33	(25)	15	(27)	75	(15)
Depression	24	(27)	22	(23)	35	(26)	52	(39)	21	(38)	154	(30)
Brief psychotic disorder	0	(0)	0	(0)	0	(0)	0	(0)	0	(0)	0	(0)
Other psychotic disorder	0	(0)	0	(0)	0	(0)	0	(0)	0	(0)	0	(0)
Adjustment disorder	2	(2)	4	(4)	1	(1)	0	(0)	1	(2)	8	(2)
Learning disability	0	(0)	0	(0)	0	(0)	0	(0)	0	(0)	0	(0)
Communication disorder	0	(0)	0	(0)	0	(0)	1	(1)	0	(0)	1	(0)
Attention Deficit Hyperactivity	0	(0)	0	(0)	0	(0)	0	(0)	0	(0)	0	(0)
Eating disorder	1	(1)	0	(0)	0	(0)	0	(0)	0	(0)	1	(0)
Parent–child relationship	0	(0)	0	(0)	0	(0)	0	(0)	0	(0)	0	(0)
Separation Anxiety	1	(1)	0	(0)	0	(0)	0	(0)	0	(0)	1	(0)
Other diagnosis	0	(0)	1	(1)	0	(0)	3	(2)	0	(0)	4	(1)
No main diagnosis	0	(0)	0	(0)	0	(0)	0	(0)	0	(0)	0	(0)
Somatic disorder	1	(1)	0	(0)	1	(1)	1	(1)	0	(0)	3	(1)
Personality disorder	0	(0)	0	(0)	1	(1)	2	(2)	0	(0)	3	(1)
Distress /no disorder	3	(3)	6	(6)	2	(1)	6	(5)	2	(4)	19	(4)
Missing value	8	(9)	3	(3)	2	(1)	1	(1)	1	(2)	15	(3)
Total	89	(100)	94	(100)	135	(100)	133	(100)	55	(100)	506	(100)

Principal diagnosis not recorded for 15 patients.

One fifth of patients (284/1357) had more than one diagnosed psychopathology. PTSD as primary or secondary diagnosis (n=157) most commonly coexisted with depression (84/157, 54%) or other anxiety disorder (23/157, 15%); the next most common were depression (n=146) with other anxiety disorders (29/146, 20%).

Baseline severity was recorded for 1286 of 1357 patients (Table 6). Patients older than 15 years were more likely to be considered severe at baseline, OR = 2.40 (1.89-3.03), while moderate baseline severity was more common after OCL, OR =2.06 (1.60-2.66). In a multivariate model (n=1263) including age, traumatic events, time to care, referral source, treatment location, and time period, variables associated with high baseline severity were: age>15 years, OR = 1.61 (1.23-2.10); greater number of traumatic events, OR = 1.33 (1.22-1.45); shorter time interval between the triggering event and seeking care OR= 0.79 (0.72-0.87); and being referred by a MSF or other health staff, OR= 2.22 (1.69-2.91); having home sessions was borderline significant, OR=1.32 (1.00-1.74); and pre/post OCL period (2007–2008 vs. 2009–2011) were not predictors of high baseline severity, OR =0.83 (0.62-1.12).

**Table 6:** Baseline severity level by age group and year of entry to MSF program

	**2007**		**2008**		**2009**		**2010**		**2011**		**Total**	
	**No.**	**(%)**	**No.**	**(%)**	**No.**	**(%)**	**No.**	**(%)**	**No.**	**(%)**	**No.**	**(%)**
**Patients 15 years of age and younger**												
mild	1	(2)	20	(20)	6	(2)	3	(1)	9	(6)	39	(5)
moderate	38	(69)	43	(43)	173	(63)	171	(71)	109	(78)	534	(66)
severe	16	(29)	38	(38)	94	(34)	67	(28)	22	(16)	237	(29)
Total	55	(100)	101	(100)	273	(100)	241	(100)	140	(100)	810	(100)
**Patients 16 years of age and older**												
mild	7	(9)	10	(11)	1	(1)	0	(0)	1	(2)	19	(4)
moderate	38	(48)	29	(33)	57	(43)	69	(55)	25	(54)	218	(46)
severe	35	(44)	49	(56)	75	(56)	56	(45)	20	(43)	235	(50)
Total	80	(100)	88	(100)	133	(100)	125	(100)	46	(100)	472	(100)

The median number of sessions for the period 2007–2011 was 9 (IQR=6-12), peaking in 2009 for patients older than 15 years and 2010 for those 15 years and younger. A multivariate model (n=1251) showed that adults, ß = 1.05, p <0.001; higher baseline severity, ß = 0.73, p <0.001; and the post OCL years (2009–2011), ß = 1.19, p <0.001 were all positively associated with greater number of sessions. Most patients (821/1332, 61%) received individual therapy, though children and younger adolescents were more likely than older adolescents and adults to receive family or dyad therapy (*χ*^2^ = 269.2, p<0.001). No significant association between number of sessions or therapy type and time period was noted.

Most patients either improved (n=1059, 78%) or had resolved the issue for which treatment was sought (n=63, 5%); 141 (10%) remained unchanged and 2 (1%) had worsened; therapeutic outcome information was not available for 92 (7%). Greatest proportion of improvements were noted in 2009–2010 (87-88% compared to 74-77% in other years), but in multivariate models including age, baseline severity and time period, no statistically significant associations were found with improvement or recovery and time period. Of the 14 patients diagnosed with ASD in 2009, 13 (93%) showed improvement or complete resolution. Of 169 with PTSD as the principal diagnosis the same year, 221 (91%) showed improvement or resolution; 11 (5%) showed no change and 10 (4%) were not evaluable. As a whole, those with anxiety disorders (ASD, PTSD, separation anxiety or other anxiety disorders) as principal diagnosis showed improvement (855/1005, 85%) more often than those with depression as principal diagnosis (141/183, 77%) (Fisher’s exact, p<0.01).

The majority of patients (n=1008, 74%) ended sessions by mutual agreement with the therapist; 52 (4%) were externally referred, mostly to local institutions, psychologists or psychiatrists for specialized care; 194 (14%) defaulted or were missing end of therapy information in the database. Symptoms persisted for 20% (266/1357) of patients, most notably: fear, anxiety or worrying (n=64), sadness (n=34), and enuresis (n=46).

## Discussion

This descriptive analysis of patient information at the MSF mental health program in Gaza Strip is based on records from two years prior through two and a half years after the bombardment of Gaza Strip between December 2008 and January 2009. The analysis detected proximal and distal socio-economic, symptomatic and diagnostic characteristics potentially associated with exposure to the violence of OCL.

Some background temporal changes were noted, such as a change in referral patterns, as well as the age, sex and socio-economic condition of patients. From 2007 to 2011 community, family and auto referrals became more common in contrast to institutional and health practitioner referrals; this is not unexpected as the organization becomes better known in the community and recommendations from former patients increase. That more vulnerable segments of the population (women, younger patients, and those with greater socio-economic needs) were seen in later years reflects a natural evolution of the program and the nature of informal referral networks in contrast to institutional and professional referrals. Some changes in baseline severity, diagnostic trends and number of sessions may also be associated with this socio-demographic shift.

Against the backdrop of programmatic and other temporally related changes, we noted some likely associations with the bombardment during Operation Cast Lead (OCL). We found a significant increase in new patients with low to middle to socio-economic needs immediately after the war. This constituted a temporary reversal of the longer-term pattern and suggests that exposure to severe violence or the destruction of material goods and traditional support networks could have an equalizing effect in terms of need for psychological support or tendency to seek it.

Due to increased demand and capacity after OCL, the average number of new monthly patients at the MSF program doubled for the following two and a half years. Noted changes among new patients after OCL were a prompt increases in diagnosis of acute stress disorder (ASD), followed by posttraumatic stress disorder (PTSD) in the first 6 months, and depression and other anxiety disorders thereafter (the former mostly for the older age group and the latter for younger); multiple diagnoses were also common. While trauma related stress often subsides weeks after the precipitating event, [27,28] diagnosed ASD is a recognized predictor of psychiatric sequelae [29]. PTSD, depression and other mental disorders are common in contexts of conflict and displacement, linked to cumulative lifetime traumatic events, modulated by adversity and daily life stressors [1,3,4,6,30].

In this impoverished and protracted, violent context, patients 15 years and younger had threefold the number of traumatic events per year of life compared to older patients, suggesting a more volatile environment in recent years. Yet despite continued adversity, one and two years after OCL a large proportion of new patients still identified a triggering event one or more years prior; the time frame suggesting that observed patterns were OCL related, or in some cases preceding it. While OCL may have constituted the principal traumatic event for many including late presenters, it may also have been the most poignant among a lifetime of accumulated daily stressors and traumatic events. It is also possible that the late presenting cases were at least partially due the continued stress exhausting resilience and coping mechanisms. The population of Gaza has been exposed to conflict over many years, which should have contributed to improved coping mechanisms [31]. Yet, it is evident that OCL impaired social networks and community capacities. It is possible that for many, built up resilience and coping mechanisms were not sufficient to manage stress and psychological needs [32].

Between 2009 and 2011 MSF received fewer professionally referred adults with more proximal triggering event, which together were found to be associated with high baseline severity. Perhaps consequently, new patients after the war tended to have moderate rather severe baseline condition. Additionally, barring changes in application of rating criteria by new personnel and increasing direct referrals of high severity cases elsewhere, it is possible that the war resulted in an increase in common disorders with moderate severity, or at least the likelihood of seeking support for them. PTSD and depression were also associated with substantial disability in other post-conflict settings [33,34].

While additional rigorous assessments are needed, psychotherapeutic and psychosocial interventions show a beneficial effect of addressing trauma related disorders in humanitarian settings. Of 13 randomized clinical controlled trials included in a recent meta-analysis, psychotherapy and psychosocial support showed beneficial effects on PTSD in adults, while group psychotherapy, psychosocial and school based support showed, more generally, benefits on children with internalizing disorders [4]. In Gaza, several interventions involving psychodynamic or cognitive therapeutic frameworks have shown promising results in reducing distress and enhancing coping strategies and resilience among war-affected children. More specifically, school based interventions including mediation, counseling, and psychodrama sessions provided emotional relief to the children or showed decreases in behavioral problems [9]. In our own intervention in Gaza, West Bank and other conflict settings, programmatic data review showed an overall benefit of psychotherapy and other mental health support [21-24]. Here, we present evidence of beneficial effects of psychotherapy on the vast majority of patients. We also describe a timeline and age related differences which if reproduced in other settings could be informative to personnel and agencies preparing to assist communities following intense violence in longer-term conflicts.

Several limitations apply to this study. These findings were based on routinely collected data, are not hypothesis driven and lack a control group; furthermore, some significant findings may simply be due to the large number of comparisons made. Second, despite efforts to standardize assessments, there is frequent turnover of expatriate staff in the program with implications on the consistency with which severity, diagnosis and treatment outcomes are assigned. Third, this is clinic based thus not necessarily representative of characteristics and changes at community level. Fourth, it was not possible to evaluate patient evolution from data collected, other than therapeutic outcome and adherence. Rather, analysis of temporal trends, including effects of the war on severity, symptomatology and diagnosis, are based on differences across new patients at different periods. Another potential limiting factor is that new versus repeat patient status was not recorded at individual level, although these were estimated to represent less than 5% of the total patient population. It is possible that returning patients may have different profiles than new patients. Similarly, since research was not the focus of the clinical intervention, some information available to clinicians was not systematically recorded in the patient follow-up database. In this instance symptom severity, traumatizing event timing and changes in operational capacity information would have enhanced the analysis of data and interpretability of results. Recording these in the future would be of benefit. Lastly, many unmeasured factors exist which could be confounding findings, such as specifics of referrals sources and reasons for them, community-wide socio-economic changes, alternative treatment facilities, and temporal trends due to program related changes.

While not a limitation, the high threshold and fluctuating levels of violence and trauma associated with the long standing conflict also makes it difficult to discern changes specific to one particular bombardment, albeit intense and long-lasting.

These findings are part of an operational review; they are meant to be hypothesis generating and would need to be confirmed in a properly designed longitudinal study. Thus from our findings we hypothesize that sudden mass exposure to acute violence in a protracted conflict may elicit a sustained increase in demand of mental health services. Such events may have an equalizing effect in terms of need for psychological support, which may be reflected in a broadening of socio-economic profile of care seekers. Coping mechanisms and resilience eroded by mass destruction may result in late presenters who still refer to a distant triggering event. Future studies would also benefit from independent assessment of resilience, severity and impact of care utilizing locally validated quantitative instruments.

## Conclusion

These findings suggest that mental health related humanitarian relief in protracted conflict might need to prepare for an increase in patients with changing profile over an extended period following an acute flare-up in violence. Specifically, observed trends suggest presentation of socio-economically diverse group of patients with a range of disorders starting with acute stress followed by PTSD and eventually depression and other anxiety disorders. The effects of a serious increase in violence at community wide levels do not seem to extinguish rapidly, as suggested by continued high demand for services and increasing time to treatment with long recall to triggering events.

## Abbreviations

ASD: Acute stress disorder; ß: Regression coefficient for linear regression; IQR: Inter-quartile range (25^th^-75^th^ percentile); OCL: Operation Cast Lead; OR: Odds ratio; PTSD: Posttraumatic stress disorder; *χ*^2^: Chi square test.

## Competing interests

The authors declare that they have no competing interests.

## Authors’ contributions

AEL had full access to the data of the study and takes full responsibility for the accuracy of the data analysis. AEL, GC, RFG, MRM participated in the interpretation of the results and critical revision of the manuscript. HT, AM participated in the collection and quality control of the data. All authors read and approved the final manuscript.
